# A Personalized Approach to Vitamin D Supplementation in Cardiovascular Health Beyond the Bone: An Expert Consensus by the Italian National Institute for Cardiovascular Research

**DOI:** 10.3390/nu17010115

**Published:** 2024-12-30

**Authors:** Anna Vittoria Mattioli, Francesca Coppi, Paolo Severino, Claudia Penna, Pasquale Pagliaro, Alessandra Dei Cas, Valentina Bucciarelli, Rosalinda Madonna, Cantor Tarperi, Federico Schena, Silvia Cetrullo, Tommaso Angelone, Carmine Rocca, Astrid Parenti, Alberto Palazzuoli, Alberto Margonato, Stefania Paolillo, Pasquale Perrone Filardi, Francesco Barillà, Carlo Lombardi, Marcello Pinti, Claudio Molinari, Antonio Cevese, Giuseppina Novo, Carmine Pizzi, Italo Porto, Corrado Poggesi, Sabina Gallina, Giuseppe Ambrosio, Francesco Fedele, on behalf of the Italian National Institute for Cardiovascular Research (INRC)

**Affiliations:** Istituto Nazionale per le Ricerche Cardiovascolari, 40126 Bologna, Italy; francesca.coppi@unimore.it (F.C.); paolo.severino@uniroma1.it (P.S.); claudia.penna@unito.it (C.P.); pasquale.pagliaro@unito.it (P.P.); alessandra.deicas@unipr.it (A.D.C.); valentina.bucciarelli@ospedaliriuniti.marche.it (V.B.); rosalinda.madonna@unipi.it (R.M.); cantor.tarperi@univr.it (C.T.); federico.schena@univr.it (F.S.); silvia.cetrullo@unibo.it (S.C.); tommaso.angelone@unical.it (T.A.); carmine.rocca@unical.it (C.R.); astrid.parenti@unifi.it (A.P.); alberto.palazzuoli@unisi.it (A.P.); margonato.alberto@hsr.it (A.M.); stefania.paolillo@unina.it (S.P.); pasquale.perronefilardi@unina.it (P.P.F.); francesco.barilla@uniroma2.it (F.B.); carlo.lombardi@unibs.it (C.L.); marcello.pinti@unimore.it (M.P.); claudio.molinari@med.uniupo.it (C.M.); antonio.cevese@univr.it (A.C.); giuseppina.novo@unipa.it (G.N.); carmine.pizzi@unibo.it (C.P.); italo.porto@unige.it (I.P.); corrado.poggesi@unifi.it (C.P.); sabina.gallina@unich.it (S.G.); giuseppe.ambrosio@unipg.it (G.A.); francesco.fedele@uniroma1.it (F.F.)

**Keywords:** vitamin D, cardiovascular risk, personalized supplementation, exposome, social determinants of health, randomized controlled trials

## Abstract

Vitamin D is increasingly recognized for its role in cardiovascular health beyond its well-established effects on bone metabolism. This review synthesizes findings from observational studies, interventional trials, and meta-analyses to clarify the mechanisms through which vitamin D impacts cardiovascular health, including its influence on vascular function, inflammation, and metabolic pathways. Additionally, this review emphasizes the importance of a personalized approach to vitamin D supplementation, integrating individual cardiovascular risk profiles, baseline vitamin D levels, and comorbid conditions, such as hypertension and diabetes. While current evidence supports the association between low vitamin D levels and increased cardiovascular mortality, this work contributes novel insights by proposing tailored strategies for supplementation, particularly for high-risk subgroups. Practical recommendations for implementing these strategies in clinical practice are also discussed, providing a framework for optimizing cardiovascular outcomes through individualized vitamin D management.

## 1. Introduction

Vitamin D plays an essential role in calcium and phosphate metabolism and maintains mineral homeostasis to ensure metabolic function and bone mineralization [1]. When, in the late 1960s, a specific receptor (vitamin D receptor VDR) was identified, it was clear that this molecule was a new steroid hormone. The hunt for the location of the VDR led to the identification of this receptor in many tissues and systems, including the cardiovascular system. Emerging research suggests that vitamin D deficiency may be linked to an increased risk of cardiovascular diseases (CVD) and associated mortality [2,3]. This review aims to synthesize current knowledge on the relationship between low levels of vitamin D and cardiovascular disease.

### 1.1. Vitamin D Synthesis and Function

Vitamin D is synthesized as D3 (cholecalciferol) from 7-dehydrocholesterol in the epidermis of the skin upon exposure to ultraviolet B (UVB, 290–320 nm wavelengths) radiation from sunlight. It can also be obtained from animal-derived dietary sources, fortified food, and supplements. Dietary sources of vitamin D3 are limited and include fatty fish, such as salmon, sardines, tuna, and cod liver oil. A vegetable form of this vitamin, D2 (ergocalciferol), can be assumed by other foods, mushrooms, in particular.

In addition, various products may be fortified with vitamin D, including dairy products, cereals, margarine, flour, and orange juice, depending on the country.

Vitamin D synthesis and metabolism have been extensively reviewed elsewhere [4]. Both forms D3 and D2, after synthesis or assumed by diet, are converted in the liver to 25-hydroxyvitamin D (25(OH)D), the main circulating form used to assess vitamin D status. D3 is more potent and effective in raising and maintaining adequate blood levels of 25(OH)D, which is essential for various physiological functions, including bone health, immune modulation, and calcium and phosphate metabolism. The levels for evaluating vitamin D status are reported below in Table 1.

A final hydroxylation step by 25(OH)D 1α-hydroxylase is essential to activate vitamin D in 1 α,25-dihydroxyvitamin D (1,25(OH)_2_D) or calcitriol [4,5,6]. This enzyme is mainly active in the kidneys, where a 24(OH)D-hydroxylase is also present and responsible for inactivating calcitriol. Other extrarenal tissues also express 25(OH)D 1α-hydroxylase, but the production of the active molecule does not appear to affect plasma levels. Instead, it remains mostly localized within the tissue, where the molecule exerts autocrine and paracrine actions [4,5,6,7].

### 1.2. Vitamin D as a Hormone

Vitamin D obtained through diet and sunlight exposure (as a prohormone) must undergo hydroxylation in the liver and kidneys to become the active form, 1,25(OH)_2_D. This active form acts as a steroid hormone, by regulating calcium and phosphate metabolism, modulating the immune system, and exerting effects on various other organs and systems. Its production, conversion, and widespread physiological effects reinforce its classification as a hormone. Understanding vitamin D’s hormonal role is crucial for appreciating its impact on health and for guiding appropriate supplementation and therapeutic strategies [7].

The most known effects of this signal molecule on gene expression are mediated by vitamin D receptors (VDRs), after dimerization with the retinoid-X receptor (RXR), and then the nuclear translocation of the complex [8]. Additionally, rapid, non-genomic responses further contribute to the complexity of mechanisms triggered by vitamin D [9].

The active form 1,25(OH)_2_D is essential for maintaining healthy bone structure and function by increasing the absorption of calcium and phosphate from the intestines, promoting the activity of osteoclasts, which release calcium from bones into the bloodstream, enhancing calcium reabsorption in the kidneys, reducing the excreted amount in urine.

The form 1,25(OH)_2_D directly suppresses the secretion of the parathyroid hormone (PTH), which promotes increased bone resorption, releasing more calcium into the bloodstream. By maintaining balanced PTH levels, vitamin D helps prevent excessive bone resorption and supports bone health. Beyond its classical role in calcium and phosphate metabolism, the presence of activating enzymes and receptors in various tissues indicates that vitamin D exerts a broad range of pleiotropic effects [6,8,9]. It enhances the immune system’s ability to combat pathogens by stimulating monocytes and macrophages, while simultaneously reducing inflammation [10,11] Specifically, vitamin D upregulates the expression of antimicrobial peptides, such as cathelicidin, which exhibit broad-spectrum antimicrobial properties. Furthermore, it modulates the adaptive immune response by influencing T cell differentiation and function, promoting the activity of regulatory T cells, and inhibiting the production of pro-inflammatory cytokines [10,11,12,13].

VDRs are expressed in vascular smooth muscle and endothelial cells, pericytes, fibroblasts, and cardiomyocytes, indicating that vitamin D may also influence cardiovascular health (i.e., by regulating blood pressure and reducing inflammation in the cardiovascular system) [11,14,15].

The definition of “normal” levels and what constitutes vitamin D deficiency remains contentious. Although it is widely agreed that 25(OH)D levels below 10 ng/mL (25 nmol/L) signify severe deficiency, there is no consensus on what should be considered a “normal” range. This ambiguity impacts both the understanding of vitamin D deficiency prevalence and clinical practices, affecting decisions around supplement prescriptions.

In clinical practice, the categories of levels of serum of 25(OH)D were suggested from the Italian Society for Osteoporosis, Mineral Metabolism, and Bone Diseases (SIOMMMS) [16] (Table 1).

**Table 1:** Categories of levels of serum 25(OH)D suggested from the Italian Society for Osteoporosis, Mineral Metabolism, and Bone Diseases (SIOMMMS).

Vitamin D Status	Serum 25(OH) D (ng/mL) In the General Population	Serum 25(OH)D (ng/mL) In Population at Risk (Including Patients at High Cardiovascular Risk)
Deficiency	<10	<10
Moderate deficiency	<20	<30
Optimal	20–50	30–50

Modified from [16].

**Figure 1:**
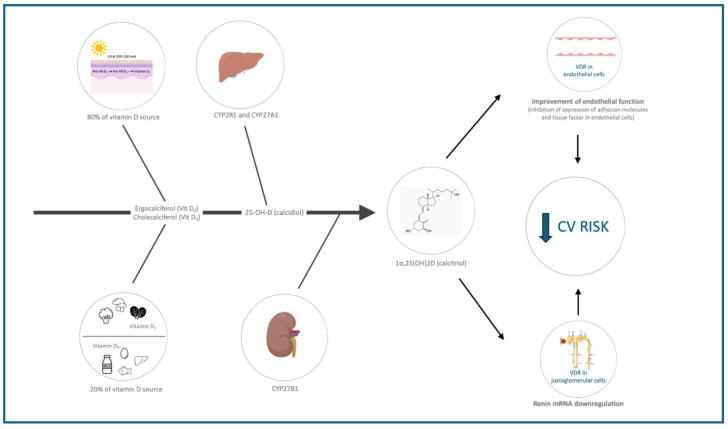
Mechanisms linking vitamin D to cardiovascular health. CV = cardiovascular, VDR = vitamin D receptor.

Vitamin D plays a crucial role in numerous cellular functions due to its ability to bind the VDR (present in various cells and organelles). The biological plausibility linking vitamin D deficiency to cardiovascular health relies on the effects of vitamin D on three key areas: endothelial function and inflammation, the renin–angiotensin–aldosterone system (RAAS), and lipid metabolism (Table 2 and Figure 1).

#### 1.2.1. Endothelial and Vascular Smooth Muscle Cell Function and Inflammation

Endothelial cells and vascular smooth muscle cells (VSMCs) express the enzyme 25(OH)D 1α-hydroxylase, in addition to VDRs, suggesting that calcitriol plays a significant role in regulating cellular function in an autocrine–paracrine manner [17]. Vitamin D has been demonstrated to improve endothelial function, which is crucial for vascular health and the prevention of atherosclerosis [17,18]. Key findings include the protection against damage induced by reactive oxygen species (ROS), particularly in human umbilical vein endothelial cells (HUVEC) with regard to proliferation and migration [14,19,20] or exposed to leptin by downregulating vascular inflammatory mediators, such as vascular cell adhesion molecule-1 (VCAM-1), monocyte chemoattractant protein-1 (MCP-1), and pro-inflammatory factors like NF-kB, and stimulating the production of nitric oxide (NO) [21].

Additionally, 1,25(OH)_2_D_3_ promotes angiogenesis through the stimulation of endothelial colony-forming cells (ECFCs), which play a critical role in blood vessel formation and repair [22]. Calcitriol also influences vascular smooth muscle cell (VSMC) function. Studies have shown that 1,25(OH)_2_D_3_ reduces the release of IL-6 and TNF-α from rat VSMCs and inhibits their proliferation in an in vitro model of vascular remodeling [23,24]. Moreover, vitamin D enhances the production of prostacyclin by VSMCs and prevents their osteogenic transdifferentiation [25]. Further evidence indicates that VSMCs isolated from vitamin D receptor knockout mice express higher levels of the angiotensin II type 1 receptor compared to those from wild-type mice, underscoring the regulatory role of vitamin D in vascular physiology [26].

#### 1.2.2. Cardiac Cells

Research suggests that vitamin D protects cardiac cells from glucose-induced damage and inhibits hypertrophy of cardiac cells triggered by angiotensin II [27,28]. Furthermore, 1,25(OH)_2_D_3_ at various concentrations has been shown to enhance the viability of cardiomyoblasts and promote their proliferation [29]. Meta-analyses of randomized controlled trials (RCTs) indicate that vitamin D supplementation reduces inflammation and oxidative stress biomarkers in individuals with diabetes. Specifically, it lowers levels of high-sensitivity C-reactive protein (hs-CRP) and malondialdehyde, while increasing nitric oxide (NO) release, total serum antioxidant capacity, and total glutathione levels [30]. Moreover, vitamin D reduces tumor necrosis factor-α (TNF-α) and inflammatory cytokines IL-1β, IL-6, and TNF-α, while promoting IL-10 secretion and M2 macrophage differentiation [31]. These findings can be seen in both healthy subjects and those with diabetes [32]. These effects reduce vascular relaxation and enhance arterial stiffness by inhibiting NO production and reducing oxidative stress.

#### 1.2.3. Renin–Angiotensin–Aldosterone System (RAAS)

Hypertension is a significant risk factor for cardiovascular disease (CVD), and vitamin D deficiency has been associated with its development. Research indicates an inverse relationship between vitamin D levels, blood pressure, and the incidence of hypertension [33,34,35]. Vitamin D plays a regulatory role in the renin–angiotensin–aldosterone system (RAAS) by suppressing renin expression, a hormone crucial for blood pressure control. By lowering renin levels, vitamin D may contribute to reducing blood pressure and mitigating the risk of hypertension [36,37]. Deficiency in 1,25(OH)_2_D_3_, the active form of vitamin D, can increase RAAS activity, leading to heightened sympathetic activity and increased intraglomerular pressure [36]. Mouse models lacking the VDR showed increased RAAS activity, elevated circulating angiotensin II and aldosterone levels, and hypertension [36]. Thus, vitamin D can be seen to negatively regulate the RAAS, and its deficiency may lead to RAAS upregulation, contributing to hypertension and cardiovascular damage.

#### 1.2.4. Lipid Metabolism

Vitamin D plays a role in lipid metabolism by potentially reducing total cholesterol, low-density lipoprotein (LDL) cholesterol, and triglycerides, while increasing high-density lipoprotein (HDL) cholesterol levels. These effects may help lower the risk of atherosclerosis [37]. Vitamin D promotes the expression of ATP-binding cassette transporter A1 (ABCA1), essential for cholesterol removal from macrophages in atherosclerotic plaque [38]. Additionally, vitamin D influences enzymes, like lipoprotein lipase and hepatic lipase, which play central roles in regulating LDL and HDL levels [39]. By modulating these enzymes, vitamin D helps maintain a proper balance between LDL and HDL, preventing atherosclerosis development and progression. Vitamin D also impacts immune response, affecting atherosclerosis progression and plaque stability [38].

#### 1.2.5. Glucose Metabolism

Vitamin D enhances insulin sensitivity and beta-cell function, potentially reducing diabetes risk. Pancreatic β cells express both VDR and the enzyme 1α-hydroxylase (Cyp27b1), which activates 25(OH)D to 1,25(OH)_3_D [39,40]. Furthermore, a vitamin D response element (VDRE) has been identified in the promoter region of the human insulin receptor gene, indicating a possible role of vitamin D in modulating insulin action, despite limited direct evidence at the INS locus [41]. In animal models, vitamin D deficiency has been linked to reduced insulin levels, while supplementation has been shown to restore islet insulin secretion [42]. Similar findings are reported in animals with VDR modifications, highlighting the role of both vitamin D and VDR in regulating insulin expression and release [43].

It is recognized that vitamin D influences glucose metabolism through at least two mechanisms: (i) binding of 1,25(OH)_3_D to the VDR induces genes involved in glucose transport, insulin secretion, and β-cell growth [41,44]; (ii) indirectly, again through VDR, it influences insulin release by modulating intracellular Ca^2+^ via activation of voltage-dependent ion channels in a PKA-dependent manner, promoting insulin exocytosis [42,45,46]. Moreover, 1,25(OH)_3_D stimulates phospholipase C (PLC) and inositol triphosphate production, which mobilizes calcium from the endoplasmic reticulum [2]. Recent research also highlights vitamin D’s role in regulating calcium via calbindin levels in human and rat pancreas [47].

Overall, vitamin D is gaining recognition as a key regulator of cardiovascular health, particularly through its impact on RAAS, where it helps modulate blood pressure and fluid balance. Intriguingly, vitamin D plays crucial roles in supporting endothelial and muscle cell function, reducing inflammation, and regulating lipid and glucose metabolism.

## 2. Observational and Interventional Insights into Vitamin D and Cardiovascular Health

### 2.1. Cohort and Cross-Sectional Studies, Observational Studies

Low vitamin D levels have been found to be associated with cardiovascular mortality. Vitamin D deficiency has emerged as a critical public health issue, with numerous studies suggesting a link between low levels of vitamin D and increased cardiovascular mortality [48,49,50,51,52]. This narrative review discusses findings from key observational studies to elucidate the association between vitamin D deficiency and cardiovascular mortality.

The synthesis of evidence from observational studies, robustly supports the association between low vitamin D levels and increased cardiovascular mortality (Supplementary Table S1). These findings highlight the potential public health implications of addressing vitamin D deficiency as a preventive strategy to reduce mortality risks but does not prove causality.

Zhang et al. explore the causal relationship between vitamin D levels and five cardiovascular conditions through Mendelian randomization [48]. Their findings establish a causal link between vitamin D levels and conditions, like angina pectoris, coronary heart disease, and lacunar stroke. However, no such relationship was identified for heart attacks or hypertension. The study employed a genome-wide association study (GWAS) combined with Mendelian randomization analysis and conducted sensitivity analyses to verify the results. The study sample consisted of 79,366 participants, all of whom had their vitamin D levels measured.

Hung et al. investigated the link between low serum 25(OH)D levels and an increased risk of cardiovascular disease (CVD). Using data from the National Health and Nutrition Examination Survey (NHANES), the study found that both vitamin D deficiency and insufficiency were associated with a higher risk of CVD. The relationship was assessed using statistical analyses and regression models, with a sample of 9825 adults aged 20 and older [49]. Similarly, Lin et al. examined the nonlinear association between higher serum 25(OH)D levels and the risk of recurrent cardiovascular events in individuals with coronary heart disease. The study suggested that maintaining sufficient vitamin D levels, particularly around 50 nmol/L, may help reduce the risk of such events. This analysis was conducted using Cox proportional hazard models and included participants from the UK Biobank [50]. In another study, Hu et al. explored the relationship between serum 25(OH)D levels and all-cause mortality in adults with a history of CVD. The findings revealed an L-shaped relationship, where increased vitamin D levels were linked to a reduction in mortality risk, though this effect plateaued at higher levels. The study used data from NHANES (2007–2018) in a cohort study, applying multivariate Cox regression models and smooth curve fitting [51]. Zhou et al. focused on the association between vitamin D levels and 10-year atherosclerotic cardiovascular disease (ASCVD) risk. The results showed that higher vitamin D levels were inversely related to ASCVD risk, underscoring the importance of maintaining adequate vitamin D levels. Cross-sectional data from NHANES (2009–2014) were analyzed, using the pooled cohort equations to estimate risk of a sample of 3354 participants [52]. In Yang et al., the research focused on the relationship between low serum 25(OH)D levels and cardiovascular risk markers. The study, conducted on a high-risk community population, confirmed a link between low vitamin D levels and increased cardiovascular risk markers and incident CVD. It was a prospective cohort study assessing both serum 25(OH)D levels and cardiovascular risk markers [53].

Another paper emphasized the significant relationship between vitamin D status and CVD. The study proposed that vitamin D levels impact cardiovascular health, partially through the downregulation of the RAAS. This study had a sample size of 10,899 participants, randomly selected from the Framingham Heart Study [54]. Kendrick et al. conducted a cross-sectional analysis using data from the third NHANES (1988–1994) to explore the relationship between serum 25(OH)D levels and the prevalence of cardiovascular disease (CVD) in a representative sample of 16,603 men and women aged 18 and older [55]. The study found a significant association between low serum 25(OH)D levels (<20 ng/mL) and a higher prevalence of CVD in the US adult population. After adjusting for confounding factors, 25(OH)D deficiency was associated with an increased risk of CVD (OR 1.20, CI 1.01–1.36, *p* = 0.03), emphasizing its independent role in CVD prevalence. In a related study, Melamed et al. also used the NHANES III database to evaluate the link between low 25(OH)D levels and mortality from all causes, cancer, and CVD in 13,331 nationally representative adults aged 20 years or older [56]. The results showed that low serum 25(OH)D levels (<17.8 ng/mL) were independently associated with a 26% higher risk of all-cause mortality (MRR 1.26, CI 1.08–1.46) over 8.7 years of follow-up. Although associations with CVD and cancer mortality were observed, they were not statistically significant, highlighting the broader health implications of 25(OH)D levels.

### 2.2. Interventional Studies and Randomized Controlled Trials (RCTs)

Despite strong observational evidence, results from RCTs and interventional studies on vitamin D supplementation and cardiovascular outcomes have yielded mixed findings. Some trials have reported no significant reduction in cardiovascular events with vitamin D supplementation, while others have shown modest benefits, particularly in individuals with baseline deficiency.

#### 2.2.1. Interventional Studies

Intervention studies [57,58,59] provide additional insights into the potential benefits and mechanisms of vitamin D on cardiovascular health (Supplementary Table S2).

The study by Desouza and colleagues examined the impact of vitamin D supplementation on cardiovascular risk in individuals with prediabetes. This was conducted as part of a secondary analysis of the D2d trial. The findings indicated that vitamin D supplementation did not result in a statistically significant reduction in the incidence of major adverse cardiovascular events (MACE) or expanded MACE when compared to a placebo. However, there was a slight improvement in the atherosclerotic cardiovascular disease (ASCVD) risk score in the vitamin D group, suggesting a potential benefit in overall cardiovascular risk reduction [57].

A study performed by Witham et al. assessed the impact of vitamin D3 supplementation (100,000 IU quarterly) on cardiovascular function in elderly patients. The study found no significant improvements in vascular health or reductions in cardiovascular events, suggesting that periodic high-dose vitamin D supplementation may not be effective in reducing cardiovascular risk in this population [58].

The VINDICATE Study evaluated the safety and efficacy of high-dose 25(OH)D3 (cholecalciferol) supplementation in patients with chronic heart failure (HF) due to left ventricular systolic dysfunction (LVSD). After one year of daily supplementation with 100 μg of vitamin D3, there was no improvement in the 6 min walk distance. However, the treatment showed beneficial effects on left ventricular structure and function in patients receiving contemporary optimal medical therapy [59].

#### 2.2.2. Randomized Controlled Trials (RCTs)

The VITamin D and omegA-3 triaL (VITAL) was a large-scale RCT aimed at evaluating the effects of vitamin D and omega-3 supplements on major cardiovascular events (Table S2). In this study, which included over 25,000 participants, vitamin D3 (2000 IU/day) supplementation did not induce significant reduction in major cardiovascular events, including heart attacks, strokes, or cardiovascular mortality, compared to the placebo group in 6-year follow-up [60]. It would be beneficial to consider the possible interactions between these factors.

While prior reviews have laid the groundwork for understanding the general role of vitamin D, our review uniquely bridges the gap between evidence and practice by proposing a practical framework for clinicians. By incorporating subgroup analyses and addressing high-risk populations, we provide a personalized approach to vitamin D supplementation, enhancing its applicability in real-world cardiovascular care.

The Vitamin D Assessment (ViDA) study was an RCT conducted in New Zealand, involving over 5000 participants who received monthly doses of vitamin D3 (100,000 IU) or a placebo for an average of 3.3 years. This study also found no significant difference in the incidence of cardiovascular events between the vitamin D and placebo groups, suggesting that high-dose vitamin D supplementation did not reduce cardiovascular event rates [61].

A substudy of the ViDA database performed by Sluyter et al. investigated the effects of 1 year of high-dose vitamin D supplementation (monthly doses equivalent to >3300 IU/day) on blood pressure parameters. It showed that, while there were no significant effects on blood pressure in the overall sample, participants with vitamin D deficiency experienced reductions in central blood pressure parameters, such as aortic systolic blood pressure and arterial stiffness. However, the study did not assess the direct outcomes of cardiovascular events [62].

In the Women’s Health Initiative (WHI) Calcium–Vitamin D Trial, which included 36,282 postmenopausal women randomized to receive calcium (1000 mg/day) plus vitamin D3 (400 IU/day) or a placebo, no significant impact of combined calcium and vitamin D supplementation was shown on coronary or cerebrovascular events; although, the dose of vitamin D used was relatively low compared to other studies [63].

The PRIMO randomized trial evaluated the effects of the active vitamin D compound, paricalcitol, on left ventricular mass over a 48-week period in patients with an estimated glomerular filtration rate (eGFR) between 15 and 60 mL/min/1.73 m^2^. The study found that 48 weeks of paricalcitol therapy did not significantly affect left ventricular mass index or improve specific measures of diastolic dysfunction in patients with chronic kidney disease [64].

The results of large RCTs, such as the VITAL study, have not always met expectations, despite robust evidence from observational studies linking low vitamin D levels with adverse cardiovascular outcomes [60]. Several potential reasons for this discrepancy warrant discussion, such as the duration of the studies, the vitamin D doses administered, the selection of participants, and the interaction between factors.

### 2.3. Meta-Analyses and Systematic Reviews

Several meta-analyses and systematic reviews have consistently highlighted the association between low vitamin D levels and an elevated risk of cardiovascular mortality (Supplementary Material Table S3). Parker et al. conducted a meta-analysis of prospective studies, finding that high vitamin D levels in middle-aged and elderly populations were significantly associated with a reduced risk of cardiovascular disease (CVD), type 2 diabetes, and metabolic syndrome. This study highlights the potential benefits of vitamin D for cardiovascular health [65].

Similarly, Schöttker et al. performed a meta-analysis of prospective cohort studies and identified a strong association between serum 25(OH)D levels and both all-cause and cardiovascular mortality in the general population. This analysis emphasizes the importance of maintaining adequate vitamin D levels to reduce mortality risks [66].

Zittermann et al. systematically reviewed observational studies, confirming that vitamin D deficiency is linked to higher mortality rates, including cardiovascular mortality. This review underscores the widespread impact of vitamin D on overall health outcomes [67].

Finally, Gaksch et al. conducted a meta-analysis of prospective studies, reinforcing the connection between low 25(OH)D levels and increased mortality, particularly from cardiovascular causes. The consistency of these findings across multiple studies strengthens the evidence, supporting the importance of vitamin D [68].

Bjelakovic et al., published in the Cochrane Database of Systematic Reviews, addressed the potential impact of vitamin D supplementation on adult mortality [69]. The review included 94,148 participants and evaluated various forms of vitamin D supplementation, including vitamin D2, vitamin D3, and active forms of vitamin D [69]. The meta-analysis found that vitamin D3 supplementation was associated with a statistically significant reduction in all-cause mortality (relative risk [RR] 0.94, 95% confidence interval [CI] 0.91 to 0.98). However, vitamin D2 and active forms of vitamin D did not show a significant effect on mortality [69].

A meta-analysis by Barbarawi et al. pooled data from 21 RCTs involving over 83,000 participants and concluded that vitamin D supplementation did not significantly reduce the risk of major adverse cardiovascular events (MACE), myocardial infarction, stroke, or cardiovascular mortality [70]. Apart from a study by Pei et al. [71], in which vitamin D supplementation was not linked to lower cardiovascular event risk, results from other meta-analyses and systematic reviews have shown that either vitamin D supplementation or low or deficient vitamin D levels were associated with cardiovascular disease events or risk factors for CVD [72,73,74,75,76,77,78]. In summary, evidence from observational, epidemiological, and interventional studies offers a nuanced perspective on the relationship between vitamin D supplementation and cardiovascular events. While these studies suggest a potential protective role of vitamin D against cardiovascular events and provide valuable insights for hypothesis generation, they do not establish causality. Most large-scale randomized controlled trials (RCTs) and meta-analyses have failed to show a significant benefit of vitamin D supplementation in reducing cardiovascular events. These inconsistencies underscore the complexity of the relationship between vitamin D and cardiovascular health, highlighting the need for more focused research to identify specific populations that may benefit from supplementation, optimal dosing strategies, and the mechanisms through which vitamin D may influence cardiovascular outcomes.

## 3. Potential Reasons for Inconsistent Findings

Several factors may contribute to the inconsistent findings between observational studies and RCTs, and these reasons are summarized below:

### 3.1. Baseline Vitamin D Status

The benefits of vitamin D supplementation tend to be more pronounced in individuals with severe deficiency compared to those with adequate or mildly deficient levels. Supplementation in individuals with severe vitamin D deficiency leads to a substantial increase in 25(OH)D levels, with noticeable improvements in health outcomes. In contrast, those with adequate or mildly deficient levels already have relatively sufficient vitamin D, so the incremental benefit of supplementation is smaller [76,77,78]. If vitamin D is primarily effective in individuals with significantly low levels, this may explain the lack of positive findings for CVD and cancer in both the ViDA and VITAL trials [60,61]. Specifically, ViDA included only 91 participants with 25(OH)D levels below 25 nmol/L, while VITAL likely had approximately 500 participants in this category, given its larger sample size and similar vitamin D distribution. Such small subgroup sizes may be insufficient to detect potential benefits for relatively uncommon outcomes, like CVD, fractures, and cancer. Additionally, null results could be due to other factors, such as the lack of combined calcium supplementation or an insufficient follow-up period to observe effects on chronic diseases. The majority of the conducted intervention studies do not assess vitamin D basal status with possible implications and limitations in the interpretation of results [60,61]. In some cases, authors collected blood samples for long-term storage, allowing later measurement of 25(OH)D to identify deficient participants, as in the case of both the ViDA and VITAL studies [60,61]. However, it remains uncertain whether institutional review boards will routinely permit this practice and for how long follow-up samples can be stored.

Furthermore, these intervention studies did not analyze the use of any vitamin supplements that the patients in the control group might have taken spontaneously. Using validated questionnaires to screen for vitamin D deficiency could be a practical alternative, but their performance tends to be only moderate. If an effective questionnaire was available, its use would differ in research versus clinical settings. Clinicians need a screening test with high sensitivity to identify most individuals with vitamin D deficiency for treatment. In contrast, clinical trials benefit more from high specificity to reduce false positives and ensure that participants are truly vitamin D deficient, thereby maximizing the positive predictive value [79,80].

Without knowing the baseline levels of vitamin D, the study cannot stratify participants based on their initial vitamin D status. This makes it difficult to determine whether the observed effects of the intervention differ between those who were severely deficient, mildly deficient, or already sufficient at the start of the study. The inability to differentiate these responses can lead to mixed or diluted results, obscuring the true impact of the intervention on different subgroups.

Furthermore, evaluating baseline levels allows researchers to establish a dose–response relationship. Without these data, it is challenging to understand how different baseline levels influence the efficacy of the intervention and whether higher doses might be necessary for those with severe deficiency.

If baseline levels are not assessed, positive results might be attributed to the intervention when, in fact, they could be due to improvements in those who were initially deficient. Conversely, a lack of observed benefits might be because the majority of participants were already sufficient in vitamin D, thereby not needing additional supplementation [60,61].

Understanding baseline levels is also crucial for assessing the safety of vitamin D supplementation. Participants with very high baseline levels might be at risk of hypercalcemia if given high doses of vitamin D, while those with very low levels might require higher doses to achieve sufficiency.

#### 3.1.1. Threshold Effects

Many biological processes influenced by vitamin D, such as calcium absorption, immune function, and bone health, have threshold effects. Severely deficient individuals are more likely to be below this critical threshold, so that supplementation may have a more immediate and significant impact by bringing their levels up to or above the threshold [16].

Adequate levels of vitamin D are essential for the efficient absorption of calcium in the intestines [81,82]. Vitamin D promotes the production of calcium-binding proteins that facilitate calcium uptake. If vitamin D levels fall below a certain threshold, calcium absorption decreases, potentially leading to calcium deficiency and its associated problems, such as weakened bones and increased fracture risk. Once the threshold is reached, calcium absorption is maximized, and additional vitamin D has little further effect on absorption efficiency [81,82].

Vitamin D is vital for the optimal functioning of the immune system [83,84,85]. It enhances the pathogen-fighting abilities of immune cells, like monocytes and macrophages, and modulates the inflammatory response. Insufficient vitamin D levels can impair immune function, leading to increased susceptibility to infections and inflammatory conditions. Reaching the necessary threshold of vitamin D is crucial for maintaining a robust immune response. Beyond this threshold, additional vitamin D does not significantly boost immune function.

Bone health is heavily reliant on vitamin D for the regulation of calcium and phosphate metabolism, which are critical for bone formation and maintenance. Adequate vitamin D levels ensure proper bone mineralization and remodeling. Below the threshold, bones may become soft and weak, leading to conditions like rickets in children and osteomalacia in adults. Meeting the threshold ensures bones are strong and healthy, but exceeding it does not provide substantial additional benefits to bone density and strength.

#### 3.1.2. Greater Potential for Correction 

Severe deficiency can lead to more pronounced clinical symptoms and health problems, such as severe bone disorders, increased risk of infections, and impaired immune response. Supplementation in severely deficient individuals can, therefore, result in more marked improvements in these symptoms and health conditions. For individuals with mild deficiency or adequate levels, the potential for noticeable clinical improvement is less significant, because they are not experiencing severe symptoms to begin with.

#### 3.1.3. Biological Compensation

The body has mechanisms to compensate for mild deficiencies in vitamin D, such as increasing the efficiency of calcium absorption in the gut and reducing renal excretion of calcium [1,2,3]. In severe deficiency, these compensatory mechanisms may be insufficient, leading to more profound physiological disturbances. Supplementation in such cases can help restore normal function and homeostasis, leading to more pronounced health benefits. The benefits of supplementation may be more pronounced in individuals with severe deficiency compared to those with adequate or mildly deficient levels.

### 3.2. Dosage and Duration

Variability in vitamin D dosage, formulation (D2 vs. D3), and the duration of supplementation in different studies may affect outcomes. Both vitamin D3 from sun exposure and dietary sources are hydroxylated in the liver to form 25(OH)D. This compound is then converted to the active form, 1,25(OH)_2_D, by the enzyme 1-alpha-hydroxylase in the kidney. Although traditionally classified as a vitamin, 1,25(OH)_2_D functions as a hormone, since it is primarily produced by one organ; although, vascular smooth muscle and endothelial cells can also convert 25(OH)D to 1,25(OH)_2_D. It exerts broad effects on multiple organs, including the cardiovascular system [1,7,16]. The level of 25(OH)D is more commonly used in clinical settings to assess vitamin D status for several reasons. First, 25(OH)D is the most abundant form of vitamin D in the bloodstream and has a longer half-life (approximately 2–3 weeks) compared to 1,25(OH)_2_D, which has a half-life of only 4–6 h [81]. This stability makes 25(OH)D a more reliable marker of an individual’s vitamin D status over time. The level of 25(OH)D reflects total vitamin D obtained from both dietary sources and sunlight exposure. Since it is produced in the liver from both vitamin D2 and vitamin D3, measuring 25(OH)D offers a comprehensive assessment of vitamin D intake from all sources.

The 1,25(OH)_2_D form of vitamin D is the biologically active form that exerts hormonal effects. Its production is tightly regulated by parathyroid hormone, calcium, and phosphate levels [86]. This regulation ensures that even with low vitamin D stores, the body can maintain normal levels of 1,25(OH)_2_D, making it less useful as an indicator of overall vitamin D status. Several studies have demonstrated that serum 25(OH)D levels are closely associated with various health outcomes, including bone health, immune function, and overall mortality. However, for more effective diagnosis and the management of vitamin D-related cardiovascular issues, it is essential to measure both 25(OH)D and 1,25(OH)_2_D. While 25(OH)D is the standard marker for assessing vitamin D status due to its longer half-life and higher serum concentration, 1,25(OH)_2_D, the active form of vitamin D, provides additional insights that can enhance diagnostic accuracy and treatment strategies [86,87]. Since 1,25(OH)_2_D exerts localized effects in tissues, such as the heart and vascular system, measuring it can offer valuable information about vitamin D’s immediate role in regulating inflammation, endothelial function, and cardiovascular health.

This 1,25(OH)_2_D form plays a critical role in reducing vascular inflammation and modulating the RAAS, both of which are crucial for managing blood pressure and preventing atherosclerosis. Measuring 1,25(OH)_2_D can guide therapy more effectively in patients with hypertension or at high risk of CVD. By measuring both 25(OH)D and 1,25(OH)_2_D, clinicians can personalize vitamin D supplementation.

In individuals with normal 25(OH)D but low 1,25(OH)_2_D, supplementation with vitamin D may be useful for optimal cardiovascular protection [86]. This strategy allows for a more personalized approach to supplementation and therapy, optimizing cardiovascular health outcomes and ensuring that patients receive appropriate treatment based on their functional vitamin D status. The recent Clinical Practice Guideline developed by the Endocrine Society concluded that there is no clinical trial evidence to support routine screening of 25(OH)D levels in the general population, including individuals with obesity or darker skin tones [7]. Instead, they concluded that, in most cases, empiric vitamin D supplementation is a cost-effective, practical, and widely acceptable approach for both healthy individuals and healthcare providers, without compromising health equity. However, the panel did not evaluate patients at CV risk but gave indications only regarding two risk factors: obesity and prediabetes. It is our opinion that screening subjects at moderate and high cardiovascular risk could improve the management of the subject and intervene on risk control as an integrative factor alongside traditional risk factors, leading to a personalized approach for management of CV risk. To summarize, 25(OH)D is the standard marker for assessing vitamin D status due to its stability and strong correlation with vitamin D stores. It is recommended for routine evaluation in populations at risk of deficiency, such as individuals with limited sun exposure, older adults, and those with chronic diseases like osteoporosis or cardiovascular conditions. On the contrary, measurement of the metabolite 1,25(OH)_2_D is generally not justified for routine use, because it reflects short-term hormonal regulation rather than overall vitamin D status. However, it may be warranted in specific clinical scenarios, such as suspected disorders of calcium metabolism or parathyroid function, chronic kidney disease, where conversion of 25(OH)D to 1,25(OH)_2_D may be impaired, and rare genetic conditions affecting vitamin D metabolism.

### 3.3. Heterogeneity of Populations Included in Studies

#### 3.3.1. Age

Differences in study populations, such as age, sex, baseline health status, and comorbidities, can significantly influence the results of studies with supplementation of vitamin D [60,61]. Age affects the absorption and metabolism of vitamin D. Older adults often have reduced the skin synthesis of vitamin D and may have decreased dietary intake. This age group might also have impaired renal function, affecting the conversion of vitamin D to its active form [87]. Hence, vitamin D supplementation may have more pronounced effects in older populations due to their higher risk of deficiency. Furthermore, since older adults are at higher risk of osteoporosis and fractures, they might show more significant benefits from supplementation compared to younger populations. The immune response weakens with age, and vitamin D plays a role in immune modulation [83,84,85]. Thus, older adults might experience enhanced immune benefits from vitamin D supplementation.

#### 3.3.2. Sex

Sex can affect the effects of vitamin D. Hormonal variations between men and women can affect vitamin D metabolism and its physiological impact [88,89,90]. For instance, estrogen has been shown to enhance the conversion of vitamin D to its active form, which might make women respond differently to supplementation. Women, particularly post-menopausal women, are at a higher risk of bone density loss and osteoporosis [89,90]. Thus, they may benefit more from vitamin D in terms of bone health compared to men.

#### 3.3.3. Physical Activity

Due to the importance of the potential effect of physical activity in avoiding vitamin D deficiency, optimizing its positive impact on the body, and improving its absorption, this topic is highly current and frequently considered in several studies and existing guidelines [91,92]. In these studies, the concentration of 25(OH)D was found to be higher in those who performed more physical activity [88,91,92,93]. However, this result may be due to various factors, some of which may be confounding, such as body mass index (BMI), the general health status of the individual taken into consideration, vitamin D supplementation, and exposure or not to sunlight during sports practice. Vitamin D can be produced endogenously by the body following exposure to sunlight, a situation promoted by some sports practices, or it can be assimilated exogenously from food sources. It has been suggested that exercise may increase serum 25(OH)D concentration by stimulating its release from adipose tissue due to lipolysis [94], regardless of whether it occurred indoors or outdoors [95]; although, the topic is still controversial [96].

Vitamin D has different absorption curves depending on the conditions in which the organism is located; the combination of lifestyle and physical activity are among the factors determining these curves. However, the term “physical activity” is highly generic and should be correctly framed. The effects of physical exercise on circulating levels of 25(OH)D and on its absorption capacity depend on the type of exercise adopted and the absolute or relative intensity with which it is administered. These individual factors must, therefore, be evaluated together to obtain a complete picture specific to each different organism [97,98].

Studies that have considered relatively low-intensity continuous exercise, commonly called “aerobic exercise” or “endurance training”, such as walking or running and characterized by activities that can be continuously maintained from several minutes up to several hours, have produced contradictory results. Some studies show that this modality of exercise can significantly increase serum 25(OH)D levels [99,100,101,102,103,104], but other studies have reported opposite results [97,104,105]. In particular, prolonged physical exercise combined with vitamin D supplementation significantly increased serum 25(OH)D, while resistance training alone did not [97]. Exercise has been shown to be effective in cases of severe vitamin D deficiency (<10 ng/mL) [106]. In contrast, this type of exercise did not appear to have significant effects on serum 25(OH)D levels in overweight and obese subjects, regardless of their vitamin D nutritional status [97]. Sex disparities in the acute response to endurance exercise have been found [105]. The potential positive effect of aerobic exercise on 25(OH)D concentrations has also been found in pathological populations, such as subjects with type 2 diabetes or metabolic syndrome [102,107]. In some studies, Nordic walking has been found to be effective in increasing 25(OH)D levels in elderly women with and without vitamin D supplementation [94,108]. Overall, the chronic effect of low-intensity continuous exercise on circulating 25(OH)D levels may be influenced by vitamin D status.

High-intensity interval training (HIIT) is characterized by repeated exercises at high metabolic intensity justified by a predominantly glycolytic metabolism alternating with phases of active or passive recovery at a lower intensity supported by a predominantly oxidative metabolism. Each single cycle includes a few minutes at high intensity alternating with a few minutes at low intensity repeated from 4 to 10 times. Few studies have considered this execution modality [99,109]. In particular, HIIT, in combination with the supplementation of 2000 IU/day of vitamin D3, has been shown to be effective in modulating appetite-dependent hormones, decreasing appetite, weight, body fat percentage, and BMI to allow an improvement in glucose tolerance [110,111]. Resistance training is characterized by exercises where the concentric muscle contraction required is high (65–80% of the maximum) or very high (81–95% of the maximum). Sometimes, exercises are performed at maximal or supramaximal intensity. In resistance exercise, the number of repetitions of the movement and, consequently, the duration of the muscle contraction is very short, depending on the load, ranging from a few seconds to a few hundred seconds. Resistance training significantly increased circulating 25(OH)D levels in both healthy subjects and in patients with vascular diseases with vitamin D deficiency [112,113]. In contrast, resistance training did not affect healthy young subjects and elderly subjects without vitamin D supplementation [105,112]. Again, several cofactors, such as vitamin D supplementation, season, and sun exposure, should be considered when interpreting these results.

#### 3.3.4. Diabetes

Individuals with diabetes mellitus (DM) are at a notably high risk of CVD [114]. A growing body of literature indicates a high prevalence of vitamin D insufficiency among individuals with DM, estimated at approximately 74.14% [115]. Low levels of 25(OH)D have been associated with an increased incidence of diabetes in older adults, even after adjusting for several potential confounders, as demonstrated in a recent large meta-analysis [116].

In the Nurses’ Health Study, a combined daily intake of more than 1200 mg of calcium and 800 IU of vitamin D was associated with a 33% reduction in the risk of developing T2D in a cohort of 83,779 women who had no prior history of DM, CVD, or cancer, compared to those with an intake of less than 600 mg of calcium and 400 IU of vitamin D [117].

In a large-scale study involving individuals at high risk of type 2 diabetes (T2D) who were not specifically selected for vitamin D insufficiency, supplementation with 4000 IU of vitamin D3 per day did not significantly reduce the risk of developing diabetes compared to a placebo [118]. Biases introduced after randomization can influence the assessment of vitamin D’s effectiveness in preventing diabetes in clinical trials. In the Vitamin D and Type 2 Diabetes (D2d) study, repeated measurements of serum 25(OH)D allowed researchers to examine whether varying levels of vitamin D exposure during the trial affected diabetes risk and whether this effect was influenced by the assigned treatment (vitamin D supplementation vs. placebo) [118]. A secondary analysis revealed a significant interaction between trial assignment and intratrial 25(OH)D levels in predicting diabetes risk (interaction *p* = 0.018). In participants assigned to the vitamin D group, each 25 nmol/L increase in intratrial 25(OH)D levels was associated with a 25% reduction in diabetes risk (hazard ratio [HR] 0.75, 95% CI 0.68–0.82). In contrast, the association was weaker in the placebo group (HR 0.90, 95% CI 0.80–1.02). For those treated with vitamin D, maintaining intratrial 25(OH)D levels between 100 and 124 nmol/L and ≥125 nmol/L corresponded to HRs for diabetes of 0.48 (95% CI 0.29–0.80) and 0.29 (95% CI 0.17–0.50), respectively, compared to those maintaining levels between 50 and 74 nmol/L. The authors concluded that daily vitamin D supplementation to maintain a serum 25(OH)D level ≥ 100 nmol/L may be a promising strategy to reduce the risk of diabetes in adults with prediabetes [119].

Regarding the potential of vitamin D supplementation to reduce the cardiovascular disease (CVD) risk associated with T2D, a recent meta-analysis indicated that vitamin D supplementation improves serum levels of high-density lipoprotein (HDL) and triglycerides (TG); although, it did not result in significant changes in low-density lipoprotein (LDL) and total cholesterol (TC) levels [120]. Based on these findings, correcting vitamin D deficiency in patients with T2D is recommended to achieve normal serum 25(OH)D concentrations, ideally around 60 nmol/L [121]. Higher serum 25(OH)D levels have been significantly associated with lower risks of total CVD in T2D patients, regardless of genetic susceptibility or variants in the vitamin D receptor (VDR). However, risk reductions tend to plateau at serum 25(OH)D levels around 50 nmol/L [122]. Recent recommendations from the Endocrine Society advocate for empiric vitamin D supplementation to reduce the risk of progression to diabetes in adults with high-risk prediabetes, alongside lifestyle modifications [7].

Obesity, a condition frequently associated to T2D and at high risk of CVD, is also characterized by a high prevalence of vitamin D deficiency. This deficiency is primarily attributed to volumetric dilution, where vitamin D is distributed into larger volumes of adipose tissue, serum, liver, and muscle. However, additional mechanisms contributing to vitamin D deficiency in obesity cannot be entirely excluded [123]. In obese individuals, vitamin D supplementation does not significantly ameliorate inflammatory status [124,125]. The recent recommendations of the Endocrine Society did not support routine screening for 25(OH)D levels, in adults with obesity [7]. Authors underline that, in adults with obesity, 25(OH)D thresholds that provide outcome-specific benefits have not been established in clinical trials [7].

#### 3.3.5. Hypertension

Research has increasingly linked low vitamin D levels with hypertension, proposing several mechanisms through which vitamin D might influence blood pressure, including its effects on the renin–angiotensin–aldosterone system (RAAS), inflammation, and endothelial function [15,33,36]. Studies have shown that individuals with lower serum vitamin D levels tend to have a higher prevalence of hypertension, a relationship observed across various populations and age groups. An analysis from the National Health and Nutrition Examination Survey (NHANES) demonstrated that individuals with lower 25(OH)D levels had a higher prevalence of hypertension, revealing an inverse relationship between vitamin D levels and blood pressure. Those in the lowest quartile of vitamin D were at a greater risk of developing hypertension [126]. Similarly, a meta-analysis by Kunutsor and colleagues reinforced the link between low vitamin D levels and increased hypertension risk, suggesting that supplementation might help mitigate this risk in certain populations [127,128].

While observational studies suggest an association, evidence from randomized controlled trials (RCTs) is more mixed, with varying results on whether vitamin D supplementation can effectively reduce blood pressure. The VITamin D and OmegA-3 TriaL (VITAL), a large-scale RCT, assessed the effects of vitamin D3 supplementation (2000 IU/day) on major health outcomes. While no significant reduction in blood pressure was observed in the overall population, a subset analysis indicated that individuals with baseline vitamin D deficiency might experience minor reductions in blood pressure with supplementation [60].

In another study, Witham et al. evaluated elderly patients with isolated systolic hypertension, supplementing them with high-dose vitamin D (100,000 IU every three months). No significant reductions in blood pressure were observed, but the study noted improvements in endothelial function, suggesting that vitamin D may have vascular benefits independent of its effects on blood pressure [129].

Seasonal variations in blood pressure, with higher readings during winter when sunlight exposure (and, thus, vitamin D synthesis) is lower, further support the connection between vitamin D and hypertension. Scragg et al. showed that vitamin D levels exhibit seasonal fluctuations, with lower levels in winter correlating with higher blood pressure measurements. This seasonal effect strengthens the hypothesis that vitamin D may influence blood pressure [130].

#### 3.3.6. Frailty

Frailty is associated with a higher prevalence of CVD. Frailty syndromes include sarcopenia, malnutrition, decreased mobility, and inflammation, each contributing to cardiovascular risk factors, such as hypertension, dyslipidemia, and insulin resistance. Frailty also frequently correlates with increased arterial stiffness, which impairs cardiovascular function and raises blood pressure, further intensifying cardiovascular risks. The mechanism underlying the frailty–CVD relationship involves chronic systemic inflammation, oxidative stress, and metabolic changes, all of which contribute to endothelial dysfunction. Additionally, frailty impairs the body’s response to stress, thereby increasing the risk of both acute and chronic cardiovascular complications, including coronary artery disease, strokes, and heart failure. In older adults, frailty and vitamin D deficiency frequently coexist, and both conditions independently increase the risk of cardiovascular events. Studies suggest a cyclical relationship between frailty, vitamin D deficiency, and CVD. Frail individuals with low vitamin D levels have a higher risk of cardiovascular complications, while CVD can exacerbate frailty symptoms and increase vitamin D needs. Low vitamin D contributes to muscle weakness and reduced mobility, both of which are critical components of frailty and important risk factors for cardiovascular events.

Research indicates that individuals with frailty and vitamin D deficiency are at a higher risk of cardiovascular events. A study by Pilz et al. highlighted that low vitamin D levels are associated with increased cardiovascular mortality, particularly in individuals with existing frailty, likely due to compounding effects on inflammation and physical resilience [131].

Other studies have suggested that vitamin D supplementation in frail older adults could improve physical performance and reduce cardiovascular risks by lowering blood pressure and improving lipid profiles [132]. However, randomized controlled trials are still needed to fully establish vitamin D’s role in reducing cardiovascular risk specifically among frail populations.

Studies show mixed results on the effectiveness of vitamin D supplementation in reducing cardiovascular risk, but some evidence suggests it may be beneficial in those who are both frail and vitamin D deficient. The recommended approach is to correct vitamin D deficiency (with levels generally under 20 ng/mL) to achieve a serum level of 25(OH)D closer to 30–50 ng/mL. This level is associated with optimal cardiovascular and musculoskeletal outcomes.

#### 3.3.7. Possible Interactions of Vitamin D with Other Factors

Vitamin D metabolism and efficacy can be significantly influenced by various factors, including medications and diet, which necessitate a more detailed analysis to fully understand these interactions.

Medications play a critical role in modulating vitamin D levels. For example, statins, widely used in cardiovascular care, may alter vitamin D status by affecting cholesterol metabolism, given that cholesterol is a precursor for vitamin D synthesis. Some studies suggest that statins may improve vitamin D levels, while others highlight potential variability based on individual responses [133]. Some statins may be increasing the absorption of vitamin D by stimulating the expressions of cholesterol transporters. This effect, which was shown with atorvastatin, can be studied with rosuvastatin and may open up a horizon to explain the link between statins and vitamin D [134]. Riche and coworkers suggested that the correction of vitamin D deficiency (≤20 ng/mL) can improve statin tolerance rates. They also stated that low vitamin D concentrations may be considered a modifiable risk factor for muscle-related adverse effects of statins [135].

Conversely, medications such as glucocorticoids and anticonvulsants can reduce vitamin D levels by increasing its catabolism, leading to a higher risk of deficiency [136,137].

This underscores the importance of monitoring vitamin D status in patients undergoing long-term treatment with these drugs and adjusting supplementation accordingly.

Dietary factors also have a profound influence on vitamin D metabolism. The intake of vitamin D-rich foods, such as fatty fish, fortified dairy products, and egg yolks, is essential for maintaining adequate levels, particularly in populations with limited sun exposure [138,139,140].

Additionally, magnesium intake is crucial, as it acts as a cofactor in the enzymatic activation of vitamin D. Diets deficient in magnesium may impair the conversion of vitamin D into its active form, 1,25(OH)_2_D, reducing its physiological effects. High dietary calcium, on the other hand, may influence vitamin D metabolism by affecting its role in calcium homeostasis, potentially altering the feedback mechanisms that regulate its levels [141].

Furthermore, the interaction between dietary patterns and medications must be considered. For instance, patients on vitamin D supplementation should avoid excessive calcium intake, which can exacerbate the risk of hypercalcemia, especially when combined with medications, like thiazide diuretics, that reduce calcium excretion.

Body mass index (BMI) plays a significant role in determining serum vitamin D levels. Numerous studies have demonstrated that individuals with higher BMI are more likely to have lower levels of circulating 25(OH)D, the primary marker of vitamin D status. This inverse relationship is primarily attributed to the sequestration of vitamin D in adipose tissue, which reduces its bioavailability in the bloodstream.

Obesity not only impacts the distribution of vitamin D but may also affect its metabolism [124,142].

The increased adiposity in obese individuals is associated with a greater volume of distribution for vitamin D, leading to a dilution effect. Furthermore, obesity-related inflammation may influence enzymes involved in vitamin D activation and degradation, potentially reducing its efficacy [124,143].

Clinical implications of this relationship highlight the need for personalized vitamin D supplementation in individuals with high BMI. Standard doses may be insufficient to correct deficiency in this population, necessitating higher doses or more frequent monitoring of serum 25(OH)D levels to ensure adequate levels are achieved and maintained.

Addressing BMI as a factor in vitamin D management is crucial for optimizing supplementation strategies, particularly in populations with a high prevalence of obesity, where deficiency is often more pronounced and requires targeted interventions [7].

The interplay between vitamin D, medications, and dietary factors underscores the complexity of optimizing vitamin D levels. Future studies should further elucidate these interactions to refine supplementation guidelines, particularly in patients with comorbidities or those on long-term pharmacological treatments. Greater understanding of this complex relationship can guide more effective and individualized approaches to the management of vitamin D in clinical practice.

### 3.4. Endpoints

Variability in primary endpoints, ranging from surrogate markers (e.g., blood pressure, lipid levels) to hard clinical outcomes (e.g., myocardial infarction, mortality), may account for discrepancies.

### 3.5. Clinical Implications and Future Directions

To accurately assess the effects of vitamin D supplementation, participants should be stratified based on age, sex, baseline levels, health status, and comorbidities. Customized dosing is crucial, as different populations, such as older adults or those with severe deficiency, may require higher doses. Appropriate outcome measures, like bone density for older women or immune function for autoimmune conditions, must be selected. A dual measurement approach for patients with cardiovascular disease and comorbid conditions can help tailor treatment more precisely. These key points are summarized in Box 1.

Box 1 **Implications for future study design and interpretation.**

1. Stratification: To accurately assess the effects of vitamin D supplementation, it is essential to stratify participants based on age, sex, baseline vitamin D levels, health status, and comorbidities. This allows for a clearer understanding of how different subgroups respond to supplementation.
2. Customized Dosing: Different populations may require different dosages of vitamin D. For example, older adults or those with severe deficiency might need higher doses compared to younger, healthier individuals.
3. Outcome Measures: Selecting appropriate outcome measures that reflect the specific benefits relevant to different subgroups is crucial. For instance, bone density measures might be more relevant for older women, while immune function might be a key outcome for individuals with autoimmune conditions.
4. Generalizability: Understanding how population differences affect study outcomes helps in generalizing the findings to broader populations. Without considering these factors, the study’s applicability to diverse groups is limited.
5. Dual Measurement Approach: For patients with CVD, especially those with comorbid conditions, such as CKD, diabetes, or metabolic syndrome, measuring both 25(OH)D and 1,25(OH)_2_D could help tailor treatment more precisely. This dual approach can reveal discrepancies between stored and active vitamin D, allowing for better-informed therapeutic interventions.

Vitamin D levels should be assessed in individuals at high cardiovascular risk, such as the elderly, the obese, and those with limited sun exposure. Deficiency is linked to hypertension, atherosclerosis, and an increased risk of cardiovascular events. A target range of >30–50 ng/mL is recommended, and supplementation may improve blood pressure and glycemic control and reduce CVD risk. Obese individuals and those with heart failure often require higher doses due to sequestration in fat tissue or chronic low levels. The regular monitoring of vitamin D levels is essential in managing hypertension, diabetes, heart failure, frailty, and rehabilitation outcomes for at-risk populations (Box 2).

Box 2 **Practical tips for physicians.**

1. Assessment of Vitamin D Levels in Cardiovascular Patients.

Indication: Measure serum 25(OH)D levels in individuals at high cardiovascular risk.

Target Range: Aim for >30–50 ng/mL. Levels below 30 ng/mL are considered insufficient and may require intervention.

At-Risk Populations: Elderly, obese individuals, people with darker skin, or those with limited sun exposure.

2. Vitamin D Deficiency and Cardiovascular Risk.

Deficiency Link: Low levels of vitamin D are associated with hypertension, atherosclerosis, and increased risk of heart attacks, strokes, and heart failure.

Indication: For individuals with CVD or high CV risk check and correct vitamin D deficiency and adopt a treat-to-target approach.

Follow-Up: Monitor CV risk and 25(OH)D levels every 6 months according to physician evaluation

3. Hypertension Management

Indication: In hypertensive patients, especially those with low vitamin D levels, supplementation may help lower blood pressure.

Follow-Up: Monitor blood pressure and 25(OH)D levels every 3–6 months.

4. Prediabetes and Diabetes and Cardiovascular Risk

Indication: Diabetics are at high risk of cardiovascular complications and often have low vitamin D. Supplementing may improve glycemic control and reduce CVD risk.

The recent recommendations of the Endocrine Society suggest empiric vitamin D supplementation to reduce the risk of progression to diabetes for adults with high-risk prediabetes, in addition to lifestyle modification

5. Obesity and Cardiovascular Risk

Indication: Obese individuals often exhibit lower vitamin D levels due to sequestration in fat tissues, increasing their cardiovascular risk.

Considerations: Monitor levels closely, as higher doses may be needed over extended periods

6. Heart Failure and Vitamin D

Indication: Patients with chronic heart failure often have low vitamin D levels, contributing to poor cardiac function and worse outcomes.

Supplementation: Regular vitamin D supplementation (1000–2000 IU/day) may improve cardiac muscle function and reduce inflammation.

Monitor: Reassess vitamin D levels and heart function periodically.

7. Frail Patients and Rehabilitation

Indication: Patients with frailty and patients that undergo cardiac rehabilitation often have low vitamin D levels.

Supplementation: Regular vitamin D supplementation may improve cardiac muscle function and reduce inflammation.

Monitor: Re-evaluate vitamin D levels and heart function periodically.

Compared to other recent publications [3,144], we have clarified how our approach builds upon their findings. While these reviews provide valuable insights into the general role of vitamin D in health, our manuscript advances the field by focusing on the integration of recent data to develop a personalized framework for vitamin D supplementation, particularly targeting populations with high cardiovascular risk [3,144,145]. The present review highlights the importance of subgroup analyses from randomized controlled trials and meta-analyses to refine supplementation strategies. This approach allows us to address the specific needs of patients with comorbidities, such as hypertension, diabetes, and metabolic syndrome. Additionally, we offer practical, clinically relevant recommendations tailored to patient demographics, including age, sex, and baseline vitamin D levels, ensuring that supplementation strategies are both effective and individualized. By addressing these nuanced aspects, our manuscript serves as a comprehensive and practical guide for implementing individualized vitamin D strategies in cardiovascular care, building upon and extending the insights offered by previous literature.

## 4. Adverse Effects of Vitamin D Overdosing

Vitamin D supplementation is widely acknowledged for its benefits in enhancing bone health and reducing cardiovascular risks. However, excessive intake can lead to vitamin D toxicity, a condition with serious health consequences. Overdosing on vitamin D raises calcium levels in the blood and tissues, resulting in hypercalcemia, hypercalciuria, and vascular calcification [146,147,148]. These imbalances can manifest as complications, such as kidney stones, where elevated calcium excretion in the urine increases the risk of stone formation. Persistent hypercalcemia may also promote vascular calcification, arterial stiffness, and heightened cardiovascular morbidity. Neurological symptoms, including confusion, fatigue, and in severe cases, altered mental status, are also associated with elevated calcium levels. Additionally, gastrointestinal distress, such as nausea, vomiting, and abdominal pain, is frequently reported in cases of vitamin D overdosing.

Toxic effects are most often linked to prolonged supplementation with doses exceeding 10,000 IU/day, emphasizing the importance of personalized vitamin D dosing. Such dosing should be guided by baseline levels and individual health profiles to ensure safety and efficacy. To minimize risks, healthcare providers should monitor serum 25(OH)D and calcium levels in patients receiving high-dose supplementation, avoid prescribing high doses without documented deficiency or specific medical indications, and educate patients about the safe upper intake levels, such as the recommended limit of 4000 IU/day for adults in the general population. By carefully tailoring supplementation to individual needs, the benefits of vitamin D can be maximized, while effectively mitigating the risks associated with excessive intake. As suggested by Brandenburg VM et al., future efforts to define the intervals of optimal vitamin D cardiovascular health must integrate serum phosphate, phosphaturia monitoring of thin changes in phosphate metabolism. It could indicate the cut between enough and too much vitamin D.

## 5. Conclusions

Maintaining adequate vitamin D levels appears to support cardiovascular health, with emerging evidence suggesting that it may be beneficial as a modifiable risk factor for CVD. This paper places importance and focus the value of a personalized approach to vitamin D supplementation, which tailors’ treatment to the patient’s unique baseline levels, age, lifestyle, and comorbidities. Such a targeted strategy has the potential to optimize cardiovascular outcomes more effectively than a uniform approach to supplementation.

Future research should prioritize large-scale RCTs with well-defined dosage regimens and extended follow-up periods to clarify vitamin D’s role in cardiovascular prevention. A randomized trial on patients with severe vitamin D deficiency is also needed. Additionally, mechanistic studies could further elucidate the specific pathways through which vitamin D impacts cardiovascular health. Implementing vitamin D supplementation with an emphasis on personalized, patient-centered therapy could advance clinical practices by integrating this cost-effective and accessible measure into broader preventive strategies for high-risk cardiovascular patients.

## Figures and Tables

**Table 2:** Mechanisms linking vitamin D to cardiovascular health.

Mechanism	Explanation
Anti-Inflammatory Effects	Cytokine Modulation: Vitamin D decreases the production of pro-inflammatory cytokines, while enhancing the expression of anti-inflammatory cytokines. Immune Regulation: Through its immunomodulatory effects, vitamin D has the potential to mitigate vascular inflammation and slow the progression of atherosclerotic plaques.
Glucose Metabolism	Insulin Sensitivity: Vitamin D improves insulin sensitivity and beta-cell function, which can help reduce the risk of diabetes.
Lipid Metabolism	Lipid Profile: Some studies suggest that vitamin D can favorably influence lipid profiles, though evidence is mixed.
Vascular Calcification	Calcium Regulation: Vitamin D plays a role in calcium homeostasis, which is vital for preventing vascular calcification.
Regulation of Blood Pressure	Renin–Angiotensin–Aldosterone System (RAAS): Vitamin D suppresses renin expression, which in turn can lower blood pressure. Vascular Tone: Vitamin D helps maintain endothelial function and vascular tone.

## Data Availability

Not applicable.

## Supplementary Materials

The following supporting information can be downloaded at: https://www.mdpi.com/article/10.3390/nu17010115/s1, Supplementary Table S1: Observational studies on low levels of vitamin D and cardiovascular disease; Supplementary Table S2: Interventional studies and randomized controlled trials on vitamin D supplementation and cardiovascular outcomes; Supplementary Table S3: Meta-analyses and systematic reviews evaluating the association between low vitamin D levels and an elevated risk of cardiovascular mortality.
