# Supplementary material for: A Personalized Approach to Vitamin D Supplementation in Cardiovascular Health Beyond the Bone: An Expert Consensus by the Italian National Institute for Cardiovascular Research
Source: Nutrients. 2024 Dec 30;17(1):115. doi: 10.3390/nu17010115 (PMC11722835; doi:10.3390/nu17010115)
Supplement: Supplementary file 1 [file nutrients-17-00115-s001.zip › Supplementary material def per invio.pdf]

Supplementary Table S1. Observational studies on low levels of vitamin D and cardiovascular disease

Study/Author [ref]	Insights	Results	Methods used	Population sample
Zhang [48]. Vitamin D levels and five cardiovascular diseases: A Mendelian randomization study.	Vitamin D levels and relationship with angina pectoris, coronary heart disease, and lacunar stroke, based on Mendelian randomization analysis.	Causal relationship found with angina, coronary heart disease, and stroke. No causal relationship found with heart attacks or hypertension.	Genome-wide association study (GWAS) with Mendelian randomization (MR) analysis Sensitivity analysis performed to verify MR results	Population sample size: 79,366 for vitamin D levels. Sampling method: Genome-wide association study (GWAS) for data collection.
Hung [49]. The Role of Vitamin D in Cardiovascular Diseases.	Low serum 25-hydroxyvitamin D levels are associated with increased risk of cardiovascular diseases, while optimal levels show no significant association. Vitamin D supplementation may benefit cardiovascular health.	25(OH)D deficiency associated with CVD (AOR = 1.48). 25(OH)D insufficiency also associated with CVD (AOR = 1.28).	National Health and Nutrition Examination Survey data Regression models to explore vitamin D-CVD association.	Population sample size: 9,825 adults aged 20 years and older. Sampling method: Utilized the 2015–2018 National Health and Nutrition Examination Survey.
Lin [50]. Associations of Serum 25(OH)D with Risk of Recurrent Cardiovascular Events in Individuals with Coronary Heart Disease.	Higher serum 25(OH)D levels show a non-linear association with reduced recurrent cardiovascular events risk in individuals with coronary heart disease, emphasizing the importance of maintaining adequate vitamin D status.	Higher serum 25(OH)D linked to lower recurrent cardiovascular event risk. Potential threshold around 50 nmol/L for optimal benefits.	Cox proportional hazard models used for calculating hazard ratios.	22,571 participants with CHD were included from the UK Biobank.
Hu [51]. Association between serum 25(OH)D and risk of all-cause mortality in adults with prior cardiovascular disease: a cohort study from NHANES 2007–2018	Increasing serum 25(OH)D levels in adults with prior cardiovascular disease show an L-shaped relationship with all-cause mortality risk, with no continued risk reduction above a certain threshold.	Serum 25(OH)D levels have an L-shaped relationship with mortality risk. Increasing serum 25(OH)D levels may not reduce mortality risk further.	Cohort study using NHANES data from 2007-2018. Multivariate Cox regression models, subgroup analyses, smooth curve fitting	3,220 participants with prior CVD were included in this study, with a total of 930 deaths over a median follow-up of 5.52 year

Zhou [52]. The Association Between Vitamin D Levels and the 10-Year Risk of Atherosclerotic Cardiovascular Disease	Higher serum 25(OH)D levels are associated with reduced 10-year atherosclerotic cardiovascular disease (ASCVD) risk, suggesting a negative relationship between vitamin D levels and ASCVD risk.	Serum 25(OH)D inversely associated with 10-year ASCVD risk. Vitamin D deficiency linked to increased ASCVD risk.	Cross-sectional data analysis from 2009–2014 NHANES. Pooled Cohort Equations used to estimate ASCVD risk	Population sample size: 3,354 participants. Sampling method: Cross-sectional data from NHANES 2009–2014
Yang 2021 [53].<br><br>Clinical Biochemistry Serum 25-hydroxyvitamin D, cardiovascular risk markers, and incident cardiovascular disease in a high risk community population.	Low serum 25-hydroxyvitamin D levels are associated with cardiovascular risk markers and incident cardiovascular disease in high-risk populations, suggesting a potential link between vitamin D levels and cardiovascular health	Low vitamin D linked to cardiovascular risk markers. Low vitamin D associated with incident cardiovascular disease in community.	Prospective cohort study. Serum 25-hydroxyvitamin D levels measured, cardiovascular risk markers assessed	Among 72,348 patients, there were 1898 CVD events over a median of 6.0 years.
Soh [54]. The Relationship Between Vitamin D Status and Cardiovascular Diseases.	The research suggests a significant relationship between vitamin D status and cardiovascular diseases, indicating a potential impact of vitamin D levels on cardiovascular health.	Reduced Vitamin D levels are correlated with an increased risk of cardiovascular diseases. Vitamin D downregulates Renin-Angiotensin-Aldosterone system activity, providing cardioprotection.	In vivo and in vitro studies. Observational studies	Population sample size: 10,899 participants. Sampling method: Random selection from Framingham Heart Study participants.
Kendrick J, [55]. Low 25-Hydroxyvitamin D Levels Are Associated with Increased All-Cause and Cardiovascular Mortality in the NHANES Study.	This study analyzes data from the NHANES to determine the association between low 25-hydroxyvitamin D levels and increased all-cause and cardiovascular mortality.	These results indicate a strong and independent relationship of 25(OH)D deficiency with prevalent CVD in a large sample representative of the US adult population	A cross-sectional analysis of data from the Third National Health and Nutrition Examination Survey	Population-based sample of 16,603 men and women aged 18 years or older.

Melamed ML, [56]. 25-hydroxyvitamin D levels and the risk of mortality in the general population.	This follow-up study from the NHANES III cohort investigates the relationship between serum 25-hydroxyvitamin D levels and the risk of death from cardiovascular and all causes.	The lowest quartile of 25(OH)D level (<17.8 ng/mL) is independently associated with all-cause mortality in the general population.	During a median 8.7 years of follow-up, there were 1806 deaths, including 777 from CVD	13,331 nationally representative adults 20 years or older from the Third National Health and Nutrition Examination Survey (NHANES III)

CVD = cardiovascular disease, CHD = coronary heart disease

Supplementary Table S2. Interventional studies and randomized controlled trials on vitamin D supplementation and cardiovascular outcomes

Study/Author [ref]	Study design	Participants	Intervention	Findings
D2d study [57].	Interventional	Patients with prediabetes	4000 IU vitamin D3 daily	A slight improvement in the atherosclerotic cardiovascular disease (ASCVD) risk score was observed in the vitamin D group
Witham et al. Study [58].	Interventional	Elderly patients	100,000 IU vitamin D3 quarterly	No significant improvements in vascular health or cardiovascular events
Witte [59]. VINDICATE Study.	Interventional	Patients with chronic heart failure (HF).	100 μg daily vitamin D3 (1 year)	No improvement in 6-min walk distance, but beneficial effects on left ventricle structure and function
Manson [60] VITAL Study	Randomised controlled trial (RCT)	25,000 participants	2,000 IU/day Vitamin D3 (6 years)	No significant reduction in major cardiovascular events (heart attacks, strokes, cardiovascular mortality)
Scragg [61]. ViDA Study	Randomised controlled trial (RCT)	5,000+ participants (New Zealand)	100,000 IU Vitamin D3 monthly (3.3 years)	No significant difference in cardiovascular events between vitamin D and placebo groups
Sluyter JD et al. [62].	Interventional	517 adults, aged between 50 and 84 years	monthly doses equivalent to >3,300 IU/day	Participants with vitamin D deficiency experienced reductions in central blood pressure parameters such as aortic systolic blood pressure and arterial stiffness
Women's Health Initiative (WHI) Calcium-Vitamin D Trial [63].	Randomised controlled trial (RCT)	36,282 postmenopausal women	Calcium (1,000 mg/day) + Vitamin D3 (400 IU/day)	No significant impact on coronary or cerebrovascular events
PRIMO Trial [64].	Randomised controlled trial (RCT)	Patients with chronic kidney disease (CKD)	Paricalcitol (48 weeks)	No effect on left ventricular mass or diastolic dysfunction

Supplementary Table S3. Meta-analyses and systematic reviews evaluating the association between low vitamin D levels and an elevated risk of cardiovascular mortality

Study/Author [ref]	Insights	Results
Parker J et al [65].	Evaluate the association between vitamin D levels, using 25-hydroxy vitamin D (25-OH-D) as an indicator of vitamin D status, and the presence of cardiometabolic disorders including cardiovascular disease, diabetes and metabolic syndrome.	The highest levels of serum 25OHD were associated with a 43% reduction in cardiometabolic disorders [odds ratio, OR 0.57, 95% (confidence interval CI 0.48–0.68)].
Schöttker B [66].	This meta-analysis investigates the relationship between serum 25-hydroxyvitamin D levels and all-cause and cardiovascular mortality in the general population	A 20 nmol/l increase in 25(OH)D levels was associated with an 8% lower mortality in the general elderly population
Zittermann A [67].	This prospective study and meta-analysis examine the association between Vitamin D deficiency and the risk of cardiovascular mortality.	Nonlinear decrease in mortality risk as circulating 25(OH)D increases, with optimal concentrations ~75-87.5 nmol/L
Gaksch M [68].	Prospective study and meta-analysis	This meta-analysis of prospective studies explores the relationship between 25-hydroxyvitamin D levels and the risk of mortality in the general population, including cardiovascular mortality
Bjelakovic et al. [69].	Meta-analysis	The meta-analysis found that vitamin D3 supplementation was associated with a statistically significant reduction in all-cause mortality (relative risk [RR] 0.94, 95% [CI] 0.91 to 0.98)
Barbarawi [70].	Vitamin D supplementation, based on a meta-analysis of 21 trials with over 83,000 participants, does not reduce cardiovascular disease risks or all-cause mortality compared to placebos.	Vitamin D supplementation not associated with reduced cardiovascular disease risks. No significant benefit in reducing major adverse cardiovascular events or mortality.
Rasouli MA [71].	Vitamin D deficiency is associated with increased cardiovascular disease risk, but supplementation does not significantly impact CVD prevention based on clinical trial evidence.	Vitamin D supplementation not recommended for CVD prevention in trials. Vitamin D deficiency associated with increased CVD risk and mortality.
Pei Y [72].	Vitamin D supplementation does not reduce cardiovascular events based on a meta-analysis of 18 trials with 70,278 participants,	Vitamin D supplementation not linked to lower cardiovascular event risk. No significant difference in subgroup analyses found.

	indicating no association with cardiovascular disease risk.	
de Lima Sousa [73].	Low vitamin D levels are associated with increased risk of cardiovascular events and death, as indicated by the systematic review, supporting a link between vitamin D levels and cardiovascular diseases.	Low vitamin D levels correlate with increased CVD risk. Vitamin D levels correlate with cardiovascular events and death.
de la Guía-Galipienso [74].	Vitamin D plays a role in cardiovascular health, with studies suggesting a potential link between vitamin D levels and the risk of cardiovascular disease based on the paper's title and abstract.	Vitamin D deficiency linked to cardiovascular disease risk factors. Vitamin D supplementation may improve cardiovascular health.
Ostadmohammadi V [75].	Vitamin D supplementation improves glycemic control, HDL-cholesterol, and reduces CRP levels in cardiovascular disease patients, indicating a beneficial impact on cardiovascular risk factors.	Vitamin D supplementation improved glycemic control and HDL-cholesterol levels. Vitamin D reduced C-reactive protein levels among CVD patients.
Rai V [76].	Vitamin D plays a significant role in cardiovascular diseases, with emerging evidence suggesting a potential link between vitamin D levels and the risk of cardiovascular disease.	Vitamin D deficiency linked to increased cardiovascular disease risk. Vitamin D supplementation may improve cardiovascular health.
Luo W [77].	Low serum 25-hydroxyvitamin D levels are associated with increased cardiovascular morbidity and mortality, including heart failure, myocardial infarction, and coronary heart disease, as per the meta-analysis.	Low serum 25OHD linked to increased cardiovascular events and mortality. Reduced 25OHD associated with higher risk of HF, MI, CHD.
Chowdhury R, [78].	This prospective study and meta-analysis investigate the association between Vitamin D levels and cardiovascular mortality.	Inverse associations of circulating 25-hydroxyvitamin D with risks of death due to cardiovascular disease, cancer, and other causes. Supplementation with vitamin D3 significantly reduces overall mortality among older adults.

CVD = cardiovascular disease, CHD = coronary heart disease, HF = heart failure, MI = myocardial infarction
