# Supplementary material for: A Personalized Approach to Vitamin D Supplementation in Cardiovascular Health Beyond the Bone: An Expert Consensus by the Italian National Institute for Cardiovascular Research
Source: Nutrients. 2024 Dec 30;17(1):115. doi: 10.3390/nu17010115 (PMC11722835; doi:10.3390/nu17010115)
Supplement: Supplementary file 1 [file nutrients-17-00115-s001.zip › Supplementary Material S4.pdf]

Supplementary Material S4

A literature search was performed to identify published studies specifically related to vitamin D supplementation or levels of vitamin D and risk of cardiovascular disease or specific outcomes/events or risk factors related to CVD. The search was performed only on articles published in peer-reviewed journals, to ensure the methodological quality of studies examined and conclusions drawn. A systematic electronic search was performed using the following search string on PubMed/Medline database (up to 1 November 2024): (("vitamin D" OR cholecalciferol OR 25(OH)D OR "25-dihydroxy vitamin D" OR "1,25-dihydroxyvitamin D" OR "hypovitaminosis D" OR "vitamin D supplementation")) AND ((cardiovascular OR heart OR cardiac OR coronary OR) AND ("acute myocardial infarction" OR AMI OR "coronary artery disease" OR event OR mortality or angina OR ASCVD)) NOT (("letter"[Filter] OR "case report" [Filter] OR "review" [Filter] "abstract" [Filter])) Filters: Clinical Study, Clinical Trial, Meta-Analysis, Observational Study, Randomized Controlled Trial, Systematic Review, English, Humans, from 2000 - 2024/11/1. The search included filters for meta-analyses, systematic reviews, clinical studies or clinical trials and published in English language (unless a specific article in another language was considered relevant). Reviews, letters, and abstracts were excluded from the search. The initial search returned a total of 220 distinct results, a total of 95 records were excluded after reading the title, abstract, full text, or publications that were considered irrelevant or not clinical studies. A further 65 studies were excluded as they were not full publications according to our inclusion criteria. The remaining 60 articles were considered potentially relevant for further evaluation, of which 31 studies are discussed in detail and summarised in Supplementary Table S1, Supplementary Table S2, and Supplementary Table S3.
