# Supplementary material for: A Personalized Approach to Vitamin D Supplementation in Cardiovascular Health Beyond the Bone: An Expert Consensus by the Italian National Institute for Cardiovascular Research
Source: Nutrients. 2024 Dec 30;17(1):115. doi: 10.3390/nu17010115 (PMC11722835; doi:10.3390/nu17010115)
Supplement: Supplementary file 1 [file nutrients-17-00115-s001.zip › What is Known def.pdf]

What is Known

Area	Current Knowledge
**Vitamin D Deficiency**	Vitamin D deficiency is prevalent and associated with multiple health risks, including cardiovascular disease (CVD).
**Vitamin D in CVD Prevention**	Evidence from observational studies suggests that low vitamin D levels may be linked to increased cardiovascular mortality, hypertension, and atherosclerosis.
**Supplementation Trials**	Mixed results from randomized controlled trials (RCTs); some suggest benefits in individuals with baseline deficiency, while others show minimal impact on cardiovascular outcomes.
**Mechanisms in CVD**	Vitamin D potentially lowers CVD risk by modulating inflammation, RAAS activity, lipid metabolism, and endothelial function.
**Dose and Duration Impact**	Effects of vitamin D depend on dosage, baseline deficiency, and duration, and lifestyle with optimal supplementation thresholds still under investigation.

What This Paper Adds to Current Knowledge

Area	Contribution of This Paper
**Personalized Supplementation**	Advocates for a tailored approach to vitamin D supplementation in CVD prevention, considering baseline deficiency, age, comorbidities, and lifestyle.
**Mechanistic Insights**	Expands on the roles of vitamin D in RAAS regulation, endothelial protection, and lipid metabolism specific to cardiovascular health.
**Focus on High-Risk Populations**	Emphasizes supplementation benefits for populations with severe deficiency or specific conditions like diabetes, CKD, and hypertension.
**Future Directions**	Calls for targeted RCTs and mechanistic studies to refine vitamin D dosing and determine long-term outcomes in CVD prevention.
