# Supplementary material for: A Personalized Approach to Vitamin D Supplementation in Cardiovascular Health Beyond the Bone: An Expert Consensus by the Italian National Institute for Cardiovascular Research
Source: Nutrients. 2024 Dec 30;17(1):115. doi: 10.3390/nu17010115 (PMC11722835; doi:10.3390/nu17010115)
Supplement: Supplementary file 1 [file nutrients-17-00115-s001.zip › What this paper adds def.pdf]

What This Paper Adds to Current Knowledge

Area	Contribution of This Paper
**Personalized Supplementation**	Advocates for a tailored approach to vitamin D supplementation in CVD prevention, considering baseline deficiency, age, comorbidities, and lifestyle.
**Mechanistic Insights**	Expands on the roles of vitamin D in RAAS regulation, endothelial protection, and lipid metabolism specific to cardiovascular health.
**Focus on High-Risk Populations**	Emphasizes supplementation benefits for populations with severe deficiency or specific conditions like diabetes, CKD, and hypertension.
**Future Directions**	Calls for targeted RCTs and mechanistic studies to refine vitamin D dosing, its correlates with physical activity, and determine long-term outcomes in CVD prevention.
